# Preoperative Arrhythmias Such as Atrial Fibrillation: Cardiovascular Surgery Risk Factor

**DOI:** 10.1155/2014/584918

**Published:** 2014-07-03

**Authors:** Diana Anghel, Radu Anghel, Flavia Corciova, Mihail Enache, Grigore Tinica

**Affiliations:** ^1^“Gr.T. Popa” University of Medicine and Pharmacy of Iasi, 700503 Iasi, Romania; ^2^Department of Cardiovascular Surgery, Institute of Cardiovascular Diseases of Iasi, 700503 Iasi, Romania

## Abstract

Atrial fibrillation is still the most common arrhythmia that occurs in heart surgery. However, there is few literature data on the manner in which preoperative atrial fibrillation may influence the postoperative outcome of various heart surgery procedures. The purpose of our research is to assess the effects of preoperative atrial fibrillation on patients having undergone different heart surgery procedures. The results of our research are a review of clinical data which were collected prospectively, over a 10-year period, from all the patients who had undergone heart surgery in our Institute. The study group included 1119 heart surgery patients, who were divided as follows: the preoperative AFib group (*n* = 226, 20.19%) and the sinus rhythm group (*n* = 893, 79.80%). Major postoperative complications and hospital mortality rates were analyzed. According to our statistical analysis, preoperative atrial fibrillation significantly increased the mortality risk (*P* = 0.001), the patients' mechanical ventilation needs (*P* = 0.022), the rate of occurrence of infectious complications (*P* < 0.5), the rate of occurrence of complications such as acute kidney failure (*P* = 0.012), and the time spent by the patients in the intensive care ward (*P* < 0.01). In conclusion, preoperative atrial fibrillation in heart surgery patients increases the mortality and major complication risk further to heart surgery.

## 1. Introduction

Atrial fibrillation (AFib) is among the most common heart surgery arrhythmias. This arrhythmia is a considerable long-term risk factor that influences the evolution and survival of patients having required heart surgery [1]. Also, the literature data revealed a significant connection between the patients' old age that needed such revascularization surgery and the atrial fibrillation incidence [2, 3]. Although it is well known that preoperative AFib increases the postoperative mortality of patients having undergone heart surgery, the literature provides little information on the way in which this arrhythmia impedes upon the postoperative outcome of patients having undergone such surgical procedures, considering the numerous complications that may occur in the evolution of these patients. There are also few studies of the evolution of preoperative AFib patients depending on the surgical procedure employed: coronary artery bypass grafting (CABG), simple or associated with heart valve surgery (CABG associated with heart valve surgery) [4, 5].

The purpose of our research is a retrospective assessment of the impact of preoperative arrhythmia such as atrial fibrillation on the postoperative evolution of patients who underwent two types of heart surgery (CABG and CABG associated with heart valve surgery), as opposed to sinus rhythm patients. The originality of our research is related to the fact that there are currently no available data on the extent to which preoperative AFib adversely affects the postoperative outcome of heart surgery in patients in Romania.

## 2. Materials and Methods

### 2.1. Patients

Our prospective research was based on a retrospective analysis. The patients included in the study were found in the database of the Institute of Cardiovascular Diseases of Iaşi, as they were hospitalized there between January 2000 and December 2010. Two types of heart surgery were conducted on these patients, namely, isolated coronary artery bypass (CABG) or heart valve surgery and CABG (coronary artery bypass). One thousand one hundred and nineteen patients (*n* = 1119) were included in the study and they were divided into two groups depending on their preoperative sinus rhythm: the AFib patient group (group *n*_1_ = 226 patients, that is, 20.19% of the total number of patients) and the normal sinus rhythm patient group (group *n*_2_ = 893 patients, that is, 79.80 % of the total number of patients). The patients with other types of arrhythmias or with pacemaker implants were not included in the study. All the prospective data were collected from a database of the department by a specialist data collector. When assessing the results, we considered all the postoperative complications that occurred during their hospitalization, the mortality rates, the time spent in the intensive care ward, and the whole hospitalization time. The management of the institute approved the use of the database of patients hospitalized during the period referred to, with a view to the analysis and publication of the results achieved.

### 2.2. Definitions

We classified atrial fibrillation according to the 2014th Guideline on Atrial Fibrillation of the American College of Cardiology/American Heart Association, in which it is classified by the duration of their episodes in paroxysmal AFib, persistent AFib, longstanding persistent AFib, permanent AFib, and nonvalvular AFib [6]. All coronary artery bypasses (CABG) were carried out under extracorporeal circulation by means of median sternotomy. Considering the standard surgical emergency criteria,* elective surgery* is defined as scheduled surgery, whereas* emergency surgery* is defined as surgery that cannot be postponed without being life threatening for the patient.* Renal complications* refer to postoperative kidney failure RIFLE “F” class (according to RIFLE system) [7].* Neurological complications* refer to the incidence of transient ischemic attacks or permanent stroke.* Infectious complications* refer to wound infections (requiring antibiotics or surgical therapy) and* sepsis*.* Pulmonary complications* refer to postoperative infection, tracheostomy, acute respiratory distress syndrome, and reintubation.

### 2.3. Statistical Analysis

The first stage of our statistical analysis sets the differences between the two groups of patients according to the study exclusion criteria and to the results achieved for the various variables considered.* Category variables* were compared by a *Z-test* for ratios or by Fisher's exact test [8].* Continuous variables* were compared by an* independent T-test* or by nonparametric tests such as McNemar's test and Wilcoxon's signed rank test designed to determine whether the normality assumption was violated (Tables 1 and 2). We then analyzed the effect of preoperative atrial fibrillation on the mortality rates and on the outcome of other essential variables for the postoperative evolution of patients under survey. This analysis was conducted by multiparameter* logistic regression* methods. Logistic regression is a prediction procedure in which the criterion or dependent variable (DV) has category values. The core of the procedure consists of developing a mathematical model which probabilistically associates criterion values (DV) and predictor values or independent variables (IV) in multiple logistic regression [9, 10]. Thus, we may rely on this type of logistic regression to determine the likelihood of preoperative arrhythmias to influence the postoperative evolution of the patients included in the two groups. The odds ratio (OR) is one of the most important concepts of logistic regression analysis. As already known, this value expresses the ratio between the likelihood of occurrence and the likelihood of nonoccurrence of an analyzed event and quantifies the impact of the predictor on the criterion. One should note that, whereas the likelihood values range between 0 and 1, their ratio may have values that are infinitely high or infinitely low (Table 3). Naturally, our goal is to use predictors that have the highest criterion prediction force. A series of logistic regression indicators, like, for instance, prediction deterioration when a particular predictor is missing, assessment of the significance of the weight coefficients (*B*) of each predictor, or the extent to which the* odds ratio* (*OR*) changes when a predictor is removed, may be used to identify these predictors. Despite the advantages described above, the logistic regression results should be construed restrainedly. The prediction model applies only to the predictors employed. Nevertheless, there may be predictors that were voluntarily or involuntarily disregarded. Also, the predictive capacity of the model only works within the variation limits of the existing data, which means that the predictive value of the predictors that were not included in the model is questionable [11]. The subgroup analysis depending on the type of operation and variable results related to atrial fibrillation (AFib) occurrence is shown in Table 4. It includes both the 95% confidence intervals (CI) and the statistical significance thresholds (*P*).

The Cox method is advisable for the accurate survival time modeling when covariates occur in the study or particular parameters are suggested that influence the evolution in time of the event. As concerns the *B* regression coefficients, it is important to calculate their statistical significance. In other words, the covariate influence in the model needs to be checked. If the calculated significance is not below 0.05, that covariate has a random effect, which means that it may be removed from the analysis. Regression coefficients are interpreted intuitively by their exponential value, Exp(*B*). This shows the predicted value of the altered hazard function value for a covariate increase by one unit. The Cox model is very important in practice, since it helps to identify the prognosis factors when studying the survival rates.

The statistical analysis was done using SPSS Version 19 software. The *P* < 0.05 values were considered statistically significant.

## 3. Results

We analyzed a cohort of patients (*N* = 1119), 200 (17.87%) of whom were female and 919 (82.12%) were male. The ages of the patients in group *n*_1_ ranged between 18 and 86 years, with a mean value (±1 S.D.) of 63.34 (±9.6) years, whereas the patients in group *n*_2_ were 20 to 79 years, with a mean value of 59.4 (±8.8) years. During their hospitalization, many patients had normal preoperative sinus rhythm (79.80%) (*n* = 893).  Table 1 distinguishes between the preoperative factors and patient characteristics of the two groups. As shown in this table, preoperative AFib patients were 3.94 years older, on the average, than the rest of the patients, most of whom were female. The incidence of congestive heart failure, kidney failure, chronic obstructive pulmonary diseases, and stroke was also higher in the AFib group.

AMI incidence within the 7 days preceding the surgery was higher in preoperative AFib patients. Smoking, cerebrovascular event, and COPD incidence was also higher in group *n*_1_. We found a higher incidence of surgical procedures such as coronary artery bypass with or without heart valve surgery in AFib patients as opposed to normal sinus rhythm patients. There was no statistically significant difference between the two groups as concerns arterial hypertension, history of AMI, or extracardiac arteriopathy.

The postoperative complications and hospital mortality rates of the two groups were synthesized in Table 2. According to the data in this table, the mortality rate was significantly higher in the AFib group (4% as opposed to 1.2%, where *P* < 0.05). Moreover, the time spent in the intensive care ward was significantly longer in the AFib group (group *n*_1_). Most types of postoperative complications, including low cardiac output, increased inotropic support needs, kidney failure, infectious complications, and reexploration (recurrent surgery), were significantly more common in AFib patients. Neurological complications were the only exceptions, as no significant increase was noted in this field. These results were calculated by means of logistic regression approaches and Cox methods (Table 3).

As for dichotomic variables, the mortality risk in AFib patients (group *n*_1_) was 1/0.086 = 11.62 times higher than in sinus rhythm patients (group *n*_2_) or, in other words, the mortality risk in sinus rhythm patients was 0.127 lower. The *B* coefficient value is negative both in the group without AFib (*B* = −2.061) and in the group with postoperative AFib (*B* = −2.459). These results show a negative correlation between AFib and the mortality rate. The more AFib found postoperatively is, the higher the mortality risk is (*P* = 0.002). Wald's statistics is significant (*P* < 0.05) for the independent variable (IV):, “considerable inotropic support needs” for the patients in group *n*_1_ and in group *n*_2_. The risk run by sinus rhythm patients (group *n*_1_) of having high inotropic support needs was 0.071 times lower than by the AFib group patients (group *n*_2_), in whom the risk of requiring increased inotropic support was 1/0.726 = 1.37 times higher. Considering the Exp(*B*) value which is close to 1 (Exp(*B*) = 0.726), one may argue that postoperative AFib increases the patient's risk of requiring inotropic support as compared to the group where AFib was not found (Exp(*B*) = 0.071). Also, the risk of occurrence of postoperative complications requiring extended mechanical ventilation (>24 h) was 11.116 times lower in sinus rhythm patients than in preoperative atrial fibrillation patients. The statistical analysis also revealed significant differences (*P* < 0.01) as concerns the number of days spent in the intensive care ward. More precisely, AFib patients (group *n*_1_) spent more days in intensive care than sinus rhythm patients (group *n*_2_). Wald's statistics is significant for the preoperative AFib group, as it proves that this condition may impede upon the postoperative outcome by increasing the number of days spent in the intensive care ward (Exp(*B*) = 1.47).

Table 4 shows subgroup analysis dependent on the type of surgical procedure used and on the effect of preoperative AFib on postoperative outcome. Multiparameter logistic regression analysis revealed that the type of surgical procedure employed influenced the level of inotropes required by the patients. Wald's statistics was significant both for group *n*_1_ and for group *n*_2_. The risk run by the patients in group *n*_1_ (suffering from preoperative AFib) of requiring increased inotropic support due to the use of coronary artery bypass graft surgery was 1.56 times higher than in the sinus rhythm patients (group *n*_2_) who underwent the same surgical therapy. As far as coronary artery bypass graft surgery associated with heart valve surgery is concerned, the risk run by sinus rhythm patients of requiring increased inotropic support was 11.15 times lower than the same risk run by preoperative AFib patients in whom the same surgical procedure was used. Also, the risk of infectious complications run by preoperative AFib patients was 6.57 times higher than the same risk run by sinus rhythm patients, in whom coronary artery bypass graft (CABG) surgery was carried out.

## 4. Discussions

AFib is still the most common arrhythmia that occurs in heart surgery, both preoperatively and postoperatively [12]. There are various studies conducted to monitor AFib influence on the postoperative outcome of patients who underwent heart surgery. Nevertheless, the literature includes a small number of studies that assess the effect of preoperative AFib on patients having had heart surgery [13–18].

In our research, we carefully analyzed as many of the postoperative AFib complications as possible, with our main goal being to determine the mortality risk run by these patients. Thus, we concluded that the old age of patients undergoing heart surgery may be an atrial fibrillation risk factor. In order to prevent such occurrences, whenever the patient is at risk, surgeons may employ adjuvant surgical techniques designed to significantly diminish the mortality risk and complication occurrence further to heart surgery done in patients with preoperative AFib arrhythmia. As shown by the data in Tables 2 and 3, the hospital mortality rate was higher in AFib patients than in sinus rhythm (SR) patients. Moreover, the occurrence of major postoperative complications and the number of days spent in the intensive care ward were lower in sinus rhythm (SR) patients as opposed to AFib patients, in whom all these parameters were significantly higher. There are many factors that may account for the effects we noted. Preoperative atrial fibrillation may lead to a low postoperative cardiac output, which makes it both important and necessary to carefully monitor the patient and to provide him/her with appropriate inotropic support. Our findings are in agreement with and are supported by the conclusions of previous studies on preoperative AFib patients [16, 18].

The data on short-term mortality rates in AFib patients is rather contradictory. Some studies reveal a higher hospital mortality rate, whereas others do not [16, 18, 19]. The main reason for these contradictory results is also the number of patients included in the study groups. More precisely, the groups with a higher number of patients were associated with significant hospital mortality rates, whereas the groups with a lower number of patients did not have the same outcome. This mortality rate increase may be connected with the postoperative complications that occurred and with the need for extended intensive care of AFib patients. There may also be other factors influenced by AFib. The literature data confirmed that the use of adjuvant surgical procedures in preoperative AFib patients is recommended to reduce the mortality risk and the occurrence of major postoperative complications [20]. Furthermore, the use of such therapeutic strategies involved significantly lower costs than conventional medical therapy [20]. The data collected for our research reveal higher mortality and morbidity rates unless other adjuvant surgical procedures are used in AFib patients. However, much more comprehensive specialized studies conducted on a much higher number of patients would be necessary in order to be able to gather conclusive data on the possible surgical therapeutic approaches that would improve the management and outcome of preoperative arrhythmia patients.

## 5. Conclusions

The results of our research are in agreement with the findings described in the literature and they revealed that preoperative atrial fibrillation increases the risk of mortality and major complications such as kidney failure, extended mechanical ventilation needs (for more than 24 hours), increased inotropic support needs, and a longer time spent in the intensive care ward further to heart surgery. Improved surgical therapeutic approaches by the use of adjuvant surgical strategies may prove beneficial for these patients.

## Figures and Tables

**Table 1:** Preoperative variables of sinus rhythm and atrial fibrillation patients (*n* = 1119).

Variable	Arrhythmia (FiA) Lot *n*_1_ = 226 patients	Sinus rhythm Lot *n*_2_ = 893 patients	*P* value
Median age ± SD	63.34 ± 9.608	59.40 ± 8.804	*P* < 0.001
Female sex	16.8% (38)	18.1% (162)	*P* > 0.05
Previous AMI	50.9% (115)	49,7% (444)	*P* > 0.05
Interval between AMI and operation			
1–7 days	1.7% (3)	0.8% (8)	*P* < 0.01
8–21 days	2.2% (5)	2.7% (24)	*P* > 0.05
21–90 days	14.1% (32)	12.7% (113)	
<90 days	18.0% (40)	16.2% (147)	
>90 days	33.2% (75)	32.7% (292)	
Diabetes mellitus	23.5% (53)	29.1% (260)	*P* > 0.05
HTN			
I	11.0% (25)	11.4% (79)	*P* > 0.05
II	28.3% (64)	29% (259)	
III	29.2% (66)	28.5% (255)	
Without HTN	31.4% (71)	33.5% (300)	
Smoker	35.9% (66)	29.2% (321)	*P* < 0.001
Serum creatinine > 200 *µ*mol/L	3.53% (8)	2.46% (22)	*P* < 0.05
COPD	9.3% (21)	7.7% (69)	*P* < 0.05
Extracardiac arterial disease	14.2% (32)	12.5% (112)	*P* > 0.05
Cerebrovascular events	15% (34)	10.9% (97)	*P* = 0.003 (*P* < 0.01)
Type of operation			
Emergent	6.6% (15)	3.1% (28)	*P* > 0.05
Elective	92% (208)	96.8% (864)	
EF < 30%	20.38 ± 4.749	23.28 ± 3.286	*P* > 0.05
EF = 30–50%	44.01 ± 6.459	44.60 ± 5.831	*P* > 0.05
EF > 50%	57.78 ± 6.940	60.81 ± 5.493	*P* < 0.001
Type of cardiac procedure			
CABG without valvular procedure	69% (156)	85.4% (763)	*P* < 0.001
CABG with valvular procedure	30.5% (69)	14.4% (129)	*P* < 0.001
Extracorporeal circulation time (min) (ECCT)	149.99 ± 66.54 The interval between 52 and 725	138.27 ± 52.75 The interval between 16 and 596	*P* = 0.002 (*P* < 0.01)
Aortic clamping time (min) (ACT)	102.069 ± 39.327 The interval between 0 and 278	95.31 ± 37.688 The interval between 11 and 330	*P* < 0.001
Mortality	4.0% (9)	1.2% (11)	*P* = 0.011 *P* < 0.05

AMI: acute myocardial infarction.

HTN: arterial hypertension.

COPD: chronic obstructive pulmonary disease.

EF: ejection fraction.

CABG: coronary artery bypass grafting.

**Table 2:** Postoperative evolution of preoperative arrhythmia patients as opposed to normal sinus rhythm patients (*n* = 1119).

Postoperative evolution	Arrhythmia Lot *n*_1_ = 226 patients	Sinus rhythm Lot *n*_2_ = 893 patients	*P* value
Increased inotropic support needs	75.7% (171)	63.9% (571)	*P* = 0.001
Reintervention	8.0% (18)	6.8% (61)	*P* = 0.028 *P* < 0.05
Mechanical ventilation > 24 h	7.5% (17)	5.5% (49)	*P* = 0.022 *P* < 0.05
MSOF	2.2% (5)	0.9% (8)	*P* < 0.05
Neurological complications CVA > 72 h	0.4% (1)	0.4% (4)	*P* > 0.05
Transient CVA	0%	0.9% (8)	*P* > 0.05
Coma > 24 h	0.9% (2)	0.7% (6)	*P* > 0.05
Acute renal failure	7.1% (16)	3.8% (34)	*P* = 0.012 *P* < 0.05
ICU LOS (days)	7.27 ± 5.056	5.53 ± 3.747	*P* < 0.01
Sepsis	2.7% (6)	1.1% (10)	*P* < 0.05
Mortality	4.0% (9)	1.2% (11)	*P* = 0.011 *P* < 0.05

CVA: cerebrovascular accident.

ICULOS: intensive care unit length of stay.

MSOF: multiple system organ failure.

**Table 3:** Logistic regression analysis of preoperative AFib arrhythmias and postoperative evolution.

Variables in the equation									
		*B*	S.E.	Wald	df	Sig.	Exp(*B*)	95% C.I. for EXP(*B*)	
								Lower	Upper
Mortality	No	−2.061	0.907	5.160	1	0.023	0.127	0.022	0.754
	Yes	−2.459	0.807	9.277	1	0.002	0.086	0.018	0416

Increased inotropic support needs	No	−2.652	1.079	6.036	1	0.014	0.071	0.009	0.585
	Yes	−0.321	0.185	3.008	1	0.043	0.726	0.505	1.043

Reintervention	No	0.474	0.569	0.695	1	0.405	1.607	0.527	4.901
	Yes	0.117	0.338	0.119	1	0.730	1.124	0.579	2.181

Mechanical ventilation > 24 h	No	2.408	1.045	5.309	1	0.021	11.116	1.433	86.226
	Yes	0.096	0.417	0.054	1	0.817	1.101	0.487	2.492

MSOF	No	2.765	1.174	0.031	1	0.191	6.954	1.532	49.135
	Yes	1.319	1.000	1.739	1	0.187	3.740	0.526	26.576

Neurological complications CVA_ > 72 h	No	2.908	1.147	0.043	1	0.732	1.203	1,421	16.130
	Yes	−0.153	1.169	0.017	1	0.896	0.858	0.087	8.479

Neurological complications: transient ischemic attacks	No	2.603	1.154	5.140	1	0.789	0.869	0.020	0.695
	Yes	0.362	0.155	1.356	1	0.899	0.696	0.008	0.565

Neurological complications: coma ≥ 72 h	No	−0.433	2.645	0.849	1	0.627	6.047	0.723	36.569
	Yes	1.173	1.214	0.933	1	0.334	3.231	0.299	13.495

Acute renal failure	No	−2.961	1.641	3.610	1	0.307	0.063	0.431	3.124
	Yes	−0.083	2.594	0.025	1	0.870	0.047	0.510	1.127

ICULOS (days)	Yes	0.046	0.023	3.942	1	0.047	1.47	1.001	1.096

Sepsis	No	−0.235	0.634	0.137	1	0.711	0.791	0.228	2.740
	Yes	−0.846	0.566	2.237	1	0.135	0.429	0.142	1.300

(i) Predicted probability (YES: preoperative arrhythmias such as AFib influence the patient's postoperative evolution; NO: preoperative arrhythmias do not influence the patient's postoperative evolution).

(ii) Predicted group (YES: with preoperative arrhythmias such as AFib—group *n*_1_; NO: without preoperative arrhythmias: sinus rhythm patients—group *n*_2_).

**Table 4:** Subgroup analysis depending on the type of heart surgery performed and on the extent of adverse effect of preoperative (AFib) arrhythmias on each unwanted postoperative event.

Variable	Patients lots	CABG lot				CABG + valvular procedures lot			
		(922 patients)				(198 patients)			
		OR	95% C.I. for OR		*P*	OR	95% C.I. for OR		*P*
			Lower	Upper			Lower	Upper	
In-hospital mortality	No	0.264	0.033	2.102	0.208	0	0	*·*	0.999
	Yes	0.357	0.057	2.248	0.273	0	0	*·*	0.999

Increased inotropic support	No	0.072	0.008	0.638	0.018	11.158	1.115	111.649	0.04
	Yes	0.638	0.419	0.972	0.036	1.242	0.564	2.735	0.049

Reintervention	No	1.649	0.445	6.115	0.455	0.433	0.052	3.612	0.439
	Yes	1.249	0.568	2.746	0.58	1.2	0.259	4.025	0.977

Mechanical ventilation_ > 24 h	No	1.844	0.269	12.637	0.533	6.97*E* + 27	0	—	0.999
	Yes	0.667	0.258	1.73	0.405	1.458	0.295	7.216	0.644

MSOF	No	2.10*E* + 09	0	—	1	0	0	—	0.999
	Yes	3.65	0.306	43.499	0.306	3.34*E* + 16	0	—	0.999

CVA_ > 72 h	No	8.87*E* + 08	0	—	1	—	—	—	—
	Yes	0.771	0.077	7.756	0.826	—	—	—	—

Transient CVA	No	1.032	0	—	1	—	—	—	—
	Yes	6.73*E* + 08	0	—	0.999	3.64*E* + 09	0	*·*	1

Coma_ > 72 h	No	0.475	0	—	1	—	—	—	—
	Yes	8.02*E* + 08	0	—	0.999	0	0	*·*	0.999

Acute renal failure	No	0	0	—	1	0.191	0.014	2.612	0.215
	Yes	0	0	—	1	0.161	0.01	2.634	0.2

ICULOS (days)	Yes	1.013	0.954	1.076	0.672	1.018	0.914	1.134	0.747

Sepsis	No	0.152	0.04	0.574	0.005	4.624	0.329	64.974	0.256
	Yes	0.384	0.108	1.366	0.139	0.408	0.06	2.762	0.358

(i) Predicted probability (YES: preoperative arrhythmias such as AFib influence postoperative evolution; NO: preoperative arrhythmias do not influence postoperative evolution).

(ii) Predicted group (YES: with preoperative arrhythmias such as AFib—group *n*_1_; NO: without preoperative arrhythmias: sinus rhythm patients—group *n*_2_).

## Limitations of the Study

Let us point out a series of inherent limitations of our study. Firstly, it is a retrospective research and hence we may rely rather on an association than on the causality specific to randomized controlled trials. Secondly, we were unable to do a long-term follow-up of our patients included in this research. Thirdly, our database could not specify the type of preoperative atrial fibrillation (for instance, whether it was paroxysmal, persistent, or permanent AFib). This detail might have had a different impact on the results of our analysis [21, 22].

Despite its limitations, one may consider this research useful, as it may be the starting point of further research in the field, the ultimate goal of which would be the discovery of new preventive surgical therapeutic approaches for preoperative atrial fibrillation patients who undergo heart surgery.
